# Iodoform in Toothache

**Published:** 1883-06

**Authors:** 


IODOFORM IN TOOTHACHE.—Schaff recommends iodoform on account of its gently caustic action as an anodyne application to exposed tooth-nerves. The circumstance that a single or repeated application of iodoform does not produce any irritation, much less any inflammation of the periosteum, and the double function of the remedy as a cleansing and disinfecting agent, make it especially appropriate as a caustic, particularly before the introduction of a temporary filling The author uses a paste consisting of

Iodoform powder, gr. 60
Kaolin, gr. 60
Carbolic acid, gr. 8
Glycerine, q. s.
Oil of peppermint, gtt. 10.

Triturate the iodoform, kaolin, and oil of peppermint with enough glycerine to form a thick paste.—*Deutsch Med. Zeit. No.* 12.

